# An Emerging Role for PI5P in T Cell Biology

**DOI:** 10.3389/fimmu.2013.00080

**Published:** 2013-04-02

**Authors:** Jacques A. Nunès, Geoffrey Guittard

**Affiliations:** ^1^Immunology and Cancer, UMR7258, CNRS, Centre de Recherche en Cancerologie de MarseilleMarseille, France; ^2^Immunology and Cancer, U1068, INSERM, Centre de Recherche en Cancerologie de MarseilleMarseille, France; ^3^Immunology and Cancer, Institut Paoli-CalmettesMarseille, France; ^4^Centre de Recherche en Cancerologie de Marseille, Aix-Marseille UniversityMarseille, France; ^5^Laboratory of Cellular and Molecular Biology, Center for Cancer Research, National Cancer InstituteBethesda, MD, USA

**Keywords:** PI5P, PtdIns5P, phosphoinositide, T cell signaling, Dok proteins

## Abstract

Phosphoinositides are critical regulators in cell biology. Phosphatidylinositol 4,5-bisphosphate, also known as PI(4,5)P_2_ or PIP_2_, was the first variety of phosphoinositide to enter in the T cell signaling scene. Phosphatidylinositol bis-phosphates are the substrates for different types of enzymes such as phospholipases C (β and γ isoforms) and phosphoinositide 3-kinases (PI3K class IA and IB) that are largely involved in signal transduction. However until recently, only a few studies highlighted phosphatidylinositol monophosphates as signaling molecules. This was mostly due to the difficulty of detection of some of these phosphoinositides, such as phosphatidylinositol 5-phosphate, also known as PI5P. Some compelling evidence argues for a role of PI5P in cell signaling and/or cell trafficking. Recently, we reported the detection of a PI5P increase upon TCR triggering. Here, we describe the current knowledge of the role of PI5P in T cell signaling. The future challenges that will be important to achieve in order to fully characterize the role of PI5P in T cell biology, will be discussed.

## Introduction

Phosphoinositides (PIs) are well known regulators of cell biology processes. Their polar inositol head group can be reversely phosphorylated on three different positions on the inositol ring (D3, D4, and D5). This can give rise to seven different phosphoinositides from the unphosphorylated one (PI) to the famous PI(3,4,5)P_3_ or PIP_3_. Phosphoinositides are anchored to cell membranes *via* two fatty acid chains inserted to the lipid bilayer. The membrane localization of phosphoinositides allows them to play a very important role in controlling protein localization within the cell, making them important players in cell signaling pathways.

From the 1950s to early 1980s, several research teams contributed to identify PI(4,5)P_2_ cleavage by the phospholipase C gamma (PLCγ) into Diacylglycerol (DAG) and Inositol tri-phosphate (IP_3_) (Berridge and Irvine, 1984). Subsequently these products lead to the activation of protein kinases C (PKC) and the release of Ca^2+^. These studies provided the first evidence that PIs could be of great importance for cell signaling. Later, the detection of increased level of PI(3,4,5)P_3_ upon oncogenic transformation and receptor tyrosine kinase (RTK) engagement led to the identification of the phosphoinositide 3 kinase (PI3K) enzymes. This introduced poly-phosphoinositides into many cell signaling pathways and identified a new common signaling pathway, PI3K/AKT, that is still under intense investigation (Whitman et al., 1988; Courtney et al., 2010; So and Fruman, 2012).

Until recently only few studies highlighted mono-PIs as signaling molecules in cell biology. This is mostly due to the difficult nature of detecting them (Pendaries et al., 2005). But several recent compelling studies argue for an important role of these mono-PIs in cell signaling. Among these mono-PIs, the phosphatidylinositol 5-phosphate PI5P has been the most recently identified PIs. Its late identification is mainly due to the difficulty in separating it from its close isomer PI4P in High-Performance-Liquid-Chromatography (HPLC) (Rameh et al., 1997; Sarkes and Rameh, 2010). Since then, several studies highlighted PI5P as a new potential important signaling molecule that could influence cell signaling pathways in epithelial cells after their activation (Pendaries et al., 2005; Wilcox and Hinchliffe, 2008; Grainger et al., 2012). Cell invasion by bacterial pathogens such as *Shigella* and *Salmonella* induce a high level of cellular PI5P. This increase is due to the Phosphoinositide 4-phosphatase activity of the virulence factors IpgD (*Shigella flexneri*) (Niebuhr et al., 2002) or SigD/SopB (*Salmonella* spp.) (Mason et al., 2007). PI5P has been localized in different subcellular compartments such as the plasma membrane, endoplasmic reticulum, Golgi apparatus, and the nucleus (Jones et al., 2006; Coronas et al., 2007; Sarkes and Rameh, 2010). PI5P was detected in T cells following ectopic expression of a PI(3,5)P_2_ 3-phosphatase, myotubularin-1 (MTM1) (Tronchere et al., 2004). MTM1 expression in Jurkat T cells induces a high level of cellular PI5P as detected by PI5P mass assay. Using similar methods, we were also able to detect a PI5P increase upon TCR triggering in the Hut-78 T cell line (Guittard et al., 2009). Recently, direct detection of PI5P by HPLC has been described (Sarkes and Rameh, 2010). These assays require expertise in analysis of lipids; thus these approaches are difficult to apply in cell signaling teams more familiar with protein biochemical analysis.

Here, we will discuss of the potent effects of PI5P in T cell signaling and the nature of the enzymes that could generate PI5P. We discuss identification of some direct PI5P partners (Guittard et al., 2009, 2010), and speculate about different protein domains that bind PI5P in order to dissect the potential functional role of PI5P and to design some potential probes for PI5P as has been done for PI(3,4,5)P_3_ detection with the Akt Pleckstrin Homology (PH) domain. Finally, increase in PI5P levels could be involved; not only in T cell signaling and gene transcription, but also in T cell chemotaxis (Konradt et al., 2011) and/or other cellular processes such as vesicular trafficking and chromatin rearrangement.

## PI5P Synthesis during T Cell Activation

Measuring PIs levels requires a large amount of cellular material. Thus, most of these experiments are performed in cell lines. Indeed, many T cell lines harbor mutations in the PTEN gene, which induces a high level of cellular PI(3,4,5)P_3_ that can induce a bias when studying other PIs species (Astoul et al., 2001). To detect PI5P levels in T cells, we used a wild-type PTEN human T cell line, HUT-78. By stimulating this cell line with an anti-CD3 mAb, a nearly fourfold PI5P increase was detected using a lipid mass assay (Guittard et al., 2009). This fold increase is in accordance with other reported results in response to insulin stimulation in other cell types (Sbrissa et al., 2004; Sarkes and Rameh, 2010). As TCR-induced PI5P elevations appear to be rapid (peaks at 2 min) and transient (Guittard et al., 2009), we suggest that there is a rapid recruitment of a specific lipid kinase/phosphatase to the plasma membrane upon TCR engagement, as observed previously for the class IA PI3K (Fabre et al., 2005). The enzyme or the enzymatic complex involved in the PI5P increase in T cells is still unknown.

**Figure 1:**
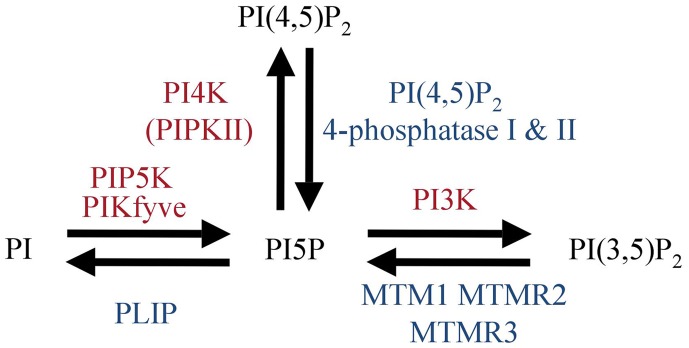
**Mammalian enzymes involved in PI5P metabolism**.

PI(4,5)P_2_ is found at high levels at the plasma membrane. Thus, it represents a potent substrate for a PI(4,5)P_2_ 4-phosphatase resulting in PI5P synthesis. IpgD *S. flexneri* virulence factor, has been clearly identified to be a PI(4,5)P_2_ 4-phosphatase (Niebuhr et al., 2002). Ectopic IpgD expression has been used in several studies to access the role of PI5P in eukaryotic cells (Pendaries et al., 2006; Guittard et al., 2009, 2010; Sarkes and Rameh, 2010; Ramel et al., 2011; Oppelt et al., 2012). So far no eukaryotic enzyme has been identified that synthesizes only PI5P. Next we will discuss enzymes that can lead to production of PI5P that are expressed in T cells and may be involved in early TCR signaling. As summarized in Figure 1, there are three different possible routes to synthesize PI5P: the 5-kinases (PIKfyve), 3-phosphatases (MTMs family members), and type I/II PI(4,5)P_2_ 4-phosphatases.

### Phosphoinositide 5-kinase, PIKfyve/PIPKIII

The simplest way to produce PI5P would be a direct phosphorylation of PI by a phosphoinositide 5-kinase. So far, only PIKfyve has been suggested to play such a role (Sbrissa et al., 1999, 2002). PIKfyve is a lipid 5-kinase that bears a FYVE domain that recognizes PI3P species. PIKfyve can act on two substrates, PI and PI3P to generate PI5P and PI(3,5)P_2_, respectively. So far, this is the only kinase proposed to directly produce PI5P from PI *in vitro*. Moreover, PIKfyve shRNAs decrease the PI5P pool in fibroblasts from a hypomorphic gene-trap mouse mutant (Zolov et al., 2012). Interestingly, their observation of the early thymus degeneration in these mice suggests a possible role for PIKfyve in T cell development. A role in peripheral T cell functions could be possible as PIKfyve is expressed in spleen (Zolov et al., 2012). However, it is still difficult to understand if the thymic degeneration result from PI5P and/or PI(3,5)P_2_ loss. Recently a new PIKfyve inhibitor, YM201636, has been identified. Unexpectedly, at low doses (10–25 nM), it inhibited preferentially PI5P rather than PI(3,5)P_2_ production *in vitro*, whereas at higher doses, the generation of the two lipid products were similarly inhibited. YM201636 or potential second generation molecules may represent a possible avenue for discriminating biologic effects observed consequent to PI5P loss versus PI(3,5)P_2_ loss (Sbrissa et al., 2012).

PI5P synthesis in different cell types occurs mainly at the plasma membrane (Sarkes and Rameh, 2010). Our observations of a rapid PI5P increase upon TCR engagement (Guittard et al., 2009) prompted us to postulate that PI5P pools are produced at the plasma membrane. However, PIKfyve is essentially located at intracellular organelles where it plays a key role in vesicular transport (Sbrissa et al., 2012). Therefore other enzymes should be considered to regulate the PI5P level at the plasma membrane.

### Phosphoinositide 3-phosphatases, MTM1, and myotubularin-related proteins

Enzymes from the myotubularin family are 3-phosphatases that can regulate PI3P and PI(3,5)P_2_ pools (Tronchere et al., 2004). These enzymes are ubiquitously expressed (Laporte et al., 1996, 1998) and are able to generate PI5P from PI(3,5)P_2_ *in vitro* (Schaletzky et al., 2003; Vaccari et al., 2011). They were the first identified eukaryotic phosphatases able to produce PI5P *in vivo* (Tronchere et al., 2004). However, here again, PI(3,5)P_2_ is thought to be mainly localized at late endosomal membranes (De Matteis and Godi, 2004). Thus, it would be difficult to consider that MTMs enzymes are responsible for early PI5P synthesis at the plasma membrane upon TCR stimulation.

### Type I/II PI(4,5)P_2_ 4-phosphatases

Two human PI(4,5)P_2_ 4-phosphatases (type I and type II isoforms) have been identified (Ungewickell et al., 2005; Zou et al., 2007). They share a CX_5_R phosphatase motif with the IpgD prokaryotic PI(4,5)P_2_ 4-phosphatase (Ungewickell et al., 2005). *In vitro*, these eukaryotic phosphatases are also able to convert PI(4,5)P_2_ to PI5P. Both enzymes are ubiquitously expressed and localize to late endosomal/lysosomal membranes in epithelial cells (Ungewickell et al., 2005). Again this makes them less likely to be involved in early signaling from the T cell receptor. Moreover type I phosphatase has been shown to be translocated to the nucleus where it can increase PI5P levels following genotoxic stress (Zou et al., 2007). Thus, this raises the possibility that these enzymes play a role in transcriptional activity.

In conclusion, based on the claimed cellular localizations of these enzymes or their substrates (Table 1), it is hard to imagine a scheme inducing a major PI5P synthesis at the plasma membrane. However, some of these enzymes have a role in T cells. For example, MTMR6 could down-regulate calcium receptor KCa3.1 expressed in T and B cells, in a PI3P-dependent manner (Srivastava et al., 2005). One cannot exclude a transient and local recruitment of enzymatic complexes able to produce PI5P at the plasma membrane. Further investigations in T cell biology studying these proteins could define some new functions for PI5P especially in endosomal compartments and/or in the nucleus.

**Table 1:** **Enzyme expression, localization, and functions in lymphoid cells**.

Enzymes	Substrates *in vitro*	Lymphoid tissues expression	Localization	Immune function	Reference
**PIPK**					
PIPKI/PI4P 5-kinase (α, β, γ)	PI4P, PI	Spl., LNs	Nu (α), PM (γ), PNu (β)	PIPKIγ in NK, Blast cells T cells, PIPKIγ 90 is negative regulator of T cell activation, adhesion, and proliferation	Doughman et al. (2003), Micucci et al. (2008), Bolomini-Vittori et al. (2009), Vasudevan et al. (2009), Wernimont et al. (2010)
PIPKIII/Pykfyve (5-kinase)	PI, PI3P	Spl., Thy	LE	KO: early degeneration of Thymus, role in T cell development?	Zolov et al. (2012)
**3-PHOSPHATASE**					
MTM1	PI3P, PI(3,5)P_2_	LN, Spl., Thy	Cyt., PM?	Overexpression enhance PI5P pool in Jurkat T cells	Laporte et al. (1996), Tronchere et al. (2004)
MTMR2, 3, 6	PI3P, PI(3,5)P_2_	Ubiquitous	Cyt., PM?	Not tested, but MTMR6 down-regulate KCa3.1 Ca2+ rec. expressed on B, T cells	Laporte et al. (1998), Walker et al. (2001), Berger et al. (2002), Schaletzky et al. (2003), Srivastava et al. (2005), Lorenzo et al. (2006), Vaccari et al. (2011)
**4-PHOSPHATASE**					
Type I, II/PI(4,5)P_2_ phosphatase (IpgD homolog)	PI(4,5)P_2_	Spl., BM, Thy., PBL	LE, Ly, Nu	Not tested	Ungewickell et al. (2005), Zou et al. (2007)
**5-PHOSPHATASE**					
PLIP/PTPM T1	PI5P?	Spl., LNs, BM	G	Not tested	Merlot et al. (2003), Pagliarini et al. (2004), Zhang et al. (2011)

*Nu, nucleus; PM, plasma membrane; C, cytosol; PNu, peri-nuclear; LE, late endosomes; Ly, lysosomes; G, Golgi; Spl, spleen; BM, bone marrow; Thy, thymus; PBL, peripheral blood leukocytes*.

## PI5P Binding Domains

As mentioned above, PIs are organized into specific subcellular compartments in order to recruit protein to a specific organelle (McCrea and De Camilli, 2009). As summarized in Table 2, some studies have been conducted to identify potential partners of PI5P and, therefore, to suggest potential functions for this phospholipid.

**Table 2:** **Some cellular proteins containing a lipid/protein interaction domain were identified as PI5P binding partners**.

Binding domain	PI binding	Experiment used	Protein role	Reference
**CYTOSOL, PLASMA MEMBRANE**				
PH Dok-1/Dok-2	PI4P, PI5P	Fat blot, SPR	Negative regulation T cell signaling	Guittard et al. (2009)
PH-Dok-4	PI5P > Mono-PIs	Fat blot, SPR	Negative/positive regulation T cell signaling	Guittard et al. (2010)
PH Dok-5	PI5P+++	Fat blot, SPR	Cardiomyocyte differentiation PI3K depdt	Guittard et al. (2010)
BIN1	PI5P, PI3P	SPR	Tubular invaginations of membranes, biogenesis of muscle T tubules	Nicot et al. (2007), Fugier et al. (2011)
**NUCLEUS**				
ATX-1-PHD	PI5P+++	Fat blot	Plant (*Arapidopsis thaliana*) chromatin modification stress induced	Alvarez-Venegas et al. (2006)
ING2 PHD	PI5P+, PI3P	Fat blot, SPR, PIP-beads	Nucleus cellular stress response	Gozani et al. (2003)
Sap30L/Sap 30	PI5P > PI3P > PI4	Fat blot	Chromatin remodeling, transcription	Viiri et al. (2009)
PH-tfb1 TFII subunit	PI5P, PI3P	Fat blot	Transcription factor	Di Lello et al. (2005)

*Different experimental approaches were used to characterize this PI5P binding. These proteins are expressed in different subcellular compartments and are involved in different cell functions. PH, Pleckstrin homology domain; PHD, plant homeo-domain; SPR, surface plasmon resonance*.

### The PHD motifs of nuclear proteins, ING2 and ATX-1

The plant homeo-domain (PHD) motif is a conserved Cys4-HisCys3 orphan zinc finger domain present throughout eukaryotic proteomes. A large number of chromatin regulatory factors contain PHD fingers, including the ING family of putative tumor suppressors (Feng et al., 2002; Fyodorov and Kadonaga, 2002; Kalkhoven et al., 2002). In 2003, the PHD of ING2 protein became the first identified PI5P-binding domain. It was identified by three different *in vitro* experimental approaches by PIP-beads binding assay, by fat-blotting, and by surface plasmon resonance (SPR) analysis (Gozani et al., 2003). However, although binding to PI5P, a significant binding to other mono-PIs such as PI3P could not be excluded from this study. To strengthen this PHD motif binding affinity, a 3X PHD motif has been generated and shows a stronger PI5P binding. This 3X PHD ING2 construct has been used as a tool for PI5P investigations (Pendaries et al., 2006; Guittard et al., 2009, 2010; Ramel et al., 2011). A similar PHD motif was identified in plants. The *Arabidopsis* homolog of trithorax-1 (ATX-1) binds PI5P using its PHD domain (Alvarez-Venegas et al., 2006). Authors are suggesting a role for PI5P in inhibiting ATX-1 protein by delocalizing it from the nucleus where it can repress gene expression. Nuclear PI5P localization has been reported and PI5P can modify the function of some PHD motif containing proteins (Gozani et al., 2003; Alvarez-Venegas et al., 2006; Jones et al., 2006). A role for nuclear PI5P in regulating nuclear protein function has not yet been assessed in T cell biology.

### The PH domain of Dok family members

Only 10% of known PH domains bind PIs with high specificity (Lemmon, 2008). The first identified PH domain harboring some PI5P binding properties was the PH domain of the p62 subunit of the transcription factor IIH (TFIIH) (Di Lello et al., 2005). This observation is really close to what has been reported for the PI5P binding PHD domains, suggesting again a potential role for PI5P in cell transcriptional activity.

Dok (for downstream of kinase) proteins are adaptor proteins that are expressed in lymphocytes (Favre et al., 2003). Upon T cell stimulation, these PH domain-containing proteins are recruited to (or are in the vicinity of) the plasma membrane (Boulay et al., 2005; Gerard et al., 2009). Dok-1 and Dok-2 PH domains were shown to bind PI5P and PI4P in SPR analysis (Guittard et al., 2009). But using different enzymatic approaches, PI5P appeared to be essential for Dok proteins tyrosine phosphorylation. Moreover, PI5P binding domain expression (Dok-1 PH domain or 3X PHD ING2) block PI5P-induced Dok phosphorylation.

Among Dok family PH domains (Favre et al., 2003), the PH domain of Dok-5 revealed the highest PI5P binding affinity in SPR experiments (Guittard et al., 2009, 2010). Dok-5 PH domain expression reduced IpgD-induced IL-2 promoter activity in T cells by sequestering PI5P within the cell (Guittard et al., 2010). These observations highlighted PI5P as a newly identified actor in T cell signaling that acts by regulating cytosolic Dok proteins.

## A Role for PI5P in T Cell Signaling

**Figure 2:**
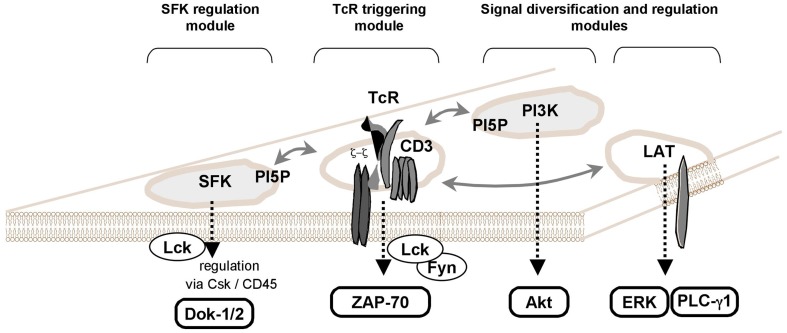
**PI5P as a new key player in TCR signaling**. TCR stimulation induces PI5P increase (Guittard et al., 2009). By expressing a bacterial PI(4,5)P_2_ 4-phosphatase, IpgD in T cells, the PI5P elevation reveals a selective activation of signaling events such as the activation of Src-family protein tyrosine kinases (SFK) and the Ser/Thr kinase, Akt (a PI3K effector) (Guittard et al., 2010). As previously illustrated (Acuto et al., 2008), some physically independent signaling modules in the T cell membranes could be involved in establishing full TCR signals, when there are interconnected. In this scheme, we added a separated module for PI3K/Akt signaling where the Class IA PI3K could recognize a SFK or some membrane protein containing a Tyr-x-x-Met motif. Plasma membrane PI5P could participate to the lipid compounds of some of these modules such as SFK regulation module and PI3K-dependent signal diversification/regulation module (see text).

Stimulation of membrane receptors such as the TCR on T cells induces the activation of protein tyrosine kinases (PTKs) and subsequently the phosphorylation of substrates, which contributes to the formation of a cytoplasmic multiprotein network (Smith-Garvin et al., 2009). TCR leads to the activation of several physically separated protein modules (Figure 2). First, Src-family protein tyrosine kinases (SFK) Lck/Fyn are activated by a yet not fully known mechanism. SFK activation is followed by tyrosine phosphorylation of TCR and CD3 chains leading to the recruitment of ZAP-70 PTK. Finally, cytoplasmic protein networks are established based on interactions with numerous adaptor proteins including LAT (Acuto et al., 2008).

Dok-5 PH expression selectively reduces some TCR-induced signaling events such as SFK activation (Lck/Fyn) and Akt phosphorylation (AKT is a PI3K effector) (Guittard et al., 2010). Independently of TCR engagement, ectopic expression of IpgD induces the phosphorylation of SFK family members and Akt (Guittard et al., 2010). Thus, PI5P could be a part of membrane signaling TCR-containing modules, such as the SFK regulation module (Acuto et al., 2008; Nika et al., 2010).

These SFKs are controlled by membrane lipid content (lipid rafts). The presence of Csk and CD45 protein tyrosine phosphatase (PTP) is involved in maintaining a balance between active and inactive SFK forms (Acuto et al., 2008). The selective inhibition of Csk activates this early SFK signaling module leading to Dok-1 tyrosine phosphorylation. This suggests that PI5P could be involved in SFK-containing lipid rafts, perhaps by modulating the dynamics of these plasma membrane structures (Schoenborn et al., 2011). Involvement of a PH containing molecule, such as SKAP-55, could also bring CD45 in close proximity to the SFK signaling module leading to its activation (Wu et al., 2002).

It has long been known that PI3K signaling is activated upon TCR triggering (Ward et al., 1992). However, it is always difficult to draw a general connection map of proximal TCR signaling pathways that integrates the PI3K/Akt pathway (Acuto et al., 2008; Smith-Garvin et al., 2009). A possible explanation would be that the PI3K/Akt signaling module is also physically independent of other proximal TCR signaling modules (Acuto et al., 2008). The full activation of Akt is dependent upon its presence in some membrane structures corresponding to lipid raft nanodomains (Lasserre et al., 2008). Several reports described PI5P acting upstream of the PI3K/Akt pathway (Carricaburu et al., 2003; Pendaries et al., 2006; Grainger et al., 2012). For instance, IpgD-produced PI5P persistently activates PI3K/Akt signaling in epithelial cells (Pendaries et al., 2006). In this condition, PI5P at the plasma membrane at the early stages of *S. flexneri* infection is rapidly enriched in endosomes and alters growth factor receptor signaling by impairing lysosomal degradation, a property used by the pathogen to favor survival of host cells. Thus far, there is no direct link between PI5P generation and PI3K activation in T cells. As it is the case for the SFK regulation module, we can hypothesize that PI5P could participate in lipid raft nanodomains dynamics where PI3K/Akt activation would take place. This potential PI3K/Akt module could also explain why PI5P elevation provokes a Dok protein tyrosine phosphorylation (Guittard et al., 2009), as the PTK Tec, a PI3K effector in T cells, phosphorylates the Dok-1 and Dok-2 proteins (Yang et al., 2001; Gerard et al., 2004).

It has been reported that PI5P and other PIs interact with high affinity to a TCR ζ basic-rich stretch (DeFord-Watts et al., 2011). The elimination of PIs-binding regions significantly impaired the ability of TCR ζ chains to be stably expressed at the plasma membrane (DeFord-Watts et al., 2011). Taken together, a role for PI5P in T cell signaling should be further investigated in these potentially physically independent modules, for instance via experiments evaluating membrane fluidity and dynamics (Lasserre et al., 2008).

## Other Perspectives for a Role of PI5P in T Cell Biology

Cell fractionation has revealed that a major fraction of PI5P is in the plasma membrane (Sarkes and Rameh, 2010). As discussed above, membrane PI5P is involved in T cell signaling (Guittard et al., 2009, 2010). This lipid could also be involved in the control of T cell migration. Indeed, *S. flexneri* is able to infect activated T cells and IpgD [converting PI(4,5)P_2_ into PI5P] inhibits chemokine-induced T cell migration (Konradt et al., 2011). In this study, the authors concluded that the T cell chemotaxis block was due to a PI(4,5)P_2_ breakdown. But they could not exclude a role for PI5P increase in this process. However, in other cell types, PI5P increases appear to induce cell migration (Oppelt et al., 2012). These apparent discrepancies likely result from differences between lymphoid cells and other cell types in their cell migration. For instance, many cell types use a PI3K-dependent pathway in inducing cell migration, but T cell chemotaxis seems to be independent of a PI3K signaling pathway (Asperti-Boursin et al., 2007). The Rac small GTPase, probably regulated by DOCK2 RacGEF, could drive this T cell migration (Fukui et al., 2001). One exiting hypothesis would be that PI5P is involved in a Rac-dependent pathway and/or participates in the connection between plasma membrane and the cytoskeleton dynamics.

In skeletal muscle, the bridging integrator-1 (BIN1) proteins bind to membrane PI5P and is involved in tubular invaginations of membranes and is required for the biogenesis of muscle T tubules (Nicot et al., 2007; Fugier et al., 2011). PI5P can be detected in endosomes (Sarkes and Rameh, 2010) and can favor RTK signaling prolongation in early endosomes (Ramel et al., 2011). Furthermore IpgD expression induces a striking amount of IL-2 promoter activity in T cells (Guittard et al., 2010). These results could be due to sustained T cell signaling at the plasma membrane or in intracellular compartments. Thus, the role of PI5P in vesicular trafficking in T cells should be considered.

In summary, PI5P is now taking its place in T cell biology. As in other mammalian cell types, the localization of basal and inducible PI5P should be characterized by cell fractionation followed by lipid composition analysis. PI5P-specific probes should be improved to visualize phospholipid dynamics upon T cell activation. Further investigations should be performed to assess the exact role of PI5P at the plasma membrane (for T cell signaling and migration), but also in vesicular trafficking and nuclear function.

## Conflict of Interest Statement

The authors declare that the research was conducted in the absence of any commercial or financial relationships that could be construed as a potential conflict of interest.
